# Long-term survival of an advanced gastric cancer patient with multiple liver metastases following maintenance therapy with envafolimab and oral chemotherapy: a case report

**DOI:** 10.3389/fonc.2025.1685259

**Published:** 2025-12-15

**Authors:** Sen Wang, Heng Zong, Linlin Wu, Yuandong Wei, Heng Wang

**Affiliations:** 1 Department of Medical Oncology, Anhui No.2 Provincial People’s Hospital, Hefei, China; 2 Department of Plastic and Reconstructive Surgery, The First Affiliated Hospital of Bengbu Medical University, Bengbu, China

**Keywords:** advanced gastric cancer, liver metastases, immunotherapy, envafolimab, raltitrexed, maintenance therapy, long-term survival

## Abstract

The survival of advanced gastric cancer with liver metastases has a grim outlook, especially in tumors which are immune cold like with HER2 negativity, PD-L1 CPS less than 1, microsatellite stable, and low tumor mutational burden. A 65-year-old patient with poorly differentiated gastric adenocarcinoma having several hepatic metastases and stage IV which stopped the first-line FOLFOX because of grade III gastrointestinal toxicity and myelosuppression is reported in this case. Molecular profiling affirmed an immune cold phenotype, and individualization of the maintenance regimen to weekly subcutaneous envafolimab and oral S-1 was induced. This was treated well and the laboratory parameters were stable thus there were no serious adverse event related to immune reactions. Serial imaging showed sustained regression and stabilization of hepatic metastases, total regression of primary lesion of the gastric lesion, and nonexistent disease. The patient had 46 months of overall survival time with a good performance status. This case recommends that maintenance therapy comprising of both PD-L1 blockage and oral fluoropyrimidine can serve as long-duration disease control and significant long-term advantage to a subset of the patients with metastatic immune-cold gastric cancer.

## Introduction

Gastric cancer is one among the most frequently found malignancies in the world, and it is mainly prevalent in the Eastern Asia region, where it has also been the leading cause of mortality due to cancer (1). The disease has a high likelihood of being at an advanced or metastatic stage when it is detected, thus high percentage is present at an advanced stage, thus with lower curability and poor survival (2). Regular chemotherapy has minimal survival advantage and even though targeted therapies and immunologic checkpoint inhibitors have enlarged the treatment, their performance as single agents still do not represent a strengths of most patients (3). In the case of metastatic gastric cancer, overall survival of the disease is normally 6 to 9 months with a 5-year survival of less than 10% despite the use of the systemic therapy (4).

The newest developments in precision oncology have spurred the combination of chemotherapy, targeted therapy, and immunotherapy to enhance outcome in a small subset of patients with PD-L1-positive or MSI-H gastric tumors (5); but again, their activity is significantly less in tumors with PD-L1 negativity and microsatellite stability (MSS) aka immune cold tumors (6, 7). Therefore, the treatment strategies using disease burden-reductive, tolerability, and sustained immunomodulation tactics should be implemented to maximize the therapeutic responses.

The presented report describes the clinical history of a patient with stage IV adenocarcinoma in the gastric area and hepatic multiple metastases with a profound level of toxicity indices during the first-line chemotherapy. The patient has had a lasting complete remission and long-term survival after changing the treatment strategy and having the combination of an oral fluoropyrimidine and a PD-L1 inhibitor. The case demonstrates the possible effect of tailored, multimodal methods of treatment to close the immune-cold gastric cancer drawbacks and enhance the overall outcomes.

## Case presentation

A 65-year-old woman reported to the clinic in October 2021 with the progressive pain in the upper abdomen, regular, intermittent distension, and intentional weight loss because she lost about 5–10 kg within a month. On physical examination, slight abdominal tenderness in the upper parts. A gastroscopy done in a local hospital showed that there was an ulcerated infiltrative lesion in the gastric fundus and biopsy showed that there was poorly differentiated adenocarcinoma.

Primary lab testing revealed high carcinoembryonic antigen (CEA, 8.04 ng/mL) and significantly impaired liver testing (ALT 73 U/L, AST 128 U/L and GGT 965 U/L). Nutritional risk screening (NRS) is 0 and the patient is in good functional condition (ECOG 0, KPS 90).

Positive results in immunohistochemistry revealed CK7, negative HER2, and high proliferative index (Ki-67 ~80%). CK20 was negative. Molecular profiling also suggested microsatellite stability (MSS), a low rate of tumor mutational burden, and expression of PD-L1 that was lower than the clinical threshold (CPS <1). Testing of Claudin18.2 was not done.

**Figure 1:**
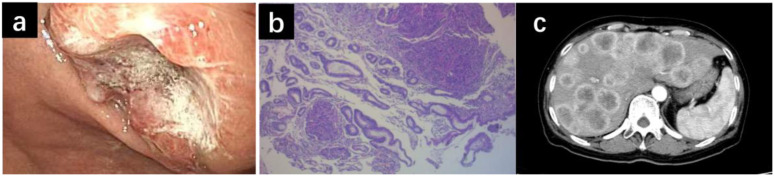
Gastroscopy revealed a new growth in the stomach **(a)**. Histopathological examination showed poorly differentiated adenocarcinoma **(b)**. CT scan showed multiple tumor metastases in the liver **(c)**.

The initial diagnosis was based on clinical, imaging, and pathological evidence and was made as stage IV gastric adenocarcinoma with multiple liver metastases and HER-2 negative (**Figure 1**).

In October 2021, the patient was initiated on the first-line chemotherapy (FOLOX). Treatment was stopped after three cycles with CTCAE grade III gastrointestinal toxicity and bone marrow suppression, along with increasing tumor markers, which was a sign of poor tolerance and poor response.

After supportive care and MDT assessment, the transition to a less intolerable long-term approach was advised. Molecular analysis showed an immune-cold pattern (HER2–, PD-L1 CPS <1, MSS, TMB-L) with little usefulness in the monotherapy of immunotherapy but possibly benefits in the combination model. Taking into account evidence that is covered by guidelines, and the lack of tolerance of the patient to 5-FU infusion, a personalized plan to use was chosen that incorporates both oral fluoropyrimidine and PD-L1 blockade.

In May 2022, the patient-initiated maintenance therapy with envafolimab (150 mg subcutaneously weekly) plus S-1 (40 mg twice daily on days 1–14 of a 21-day cycle). The regimen was well tolerated, with only mild fatigue and no grade ≥2 hematologic or immune-related adverse events. Laboratory parameters remained stable throughout treatment.

**Figure 2:**
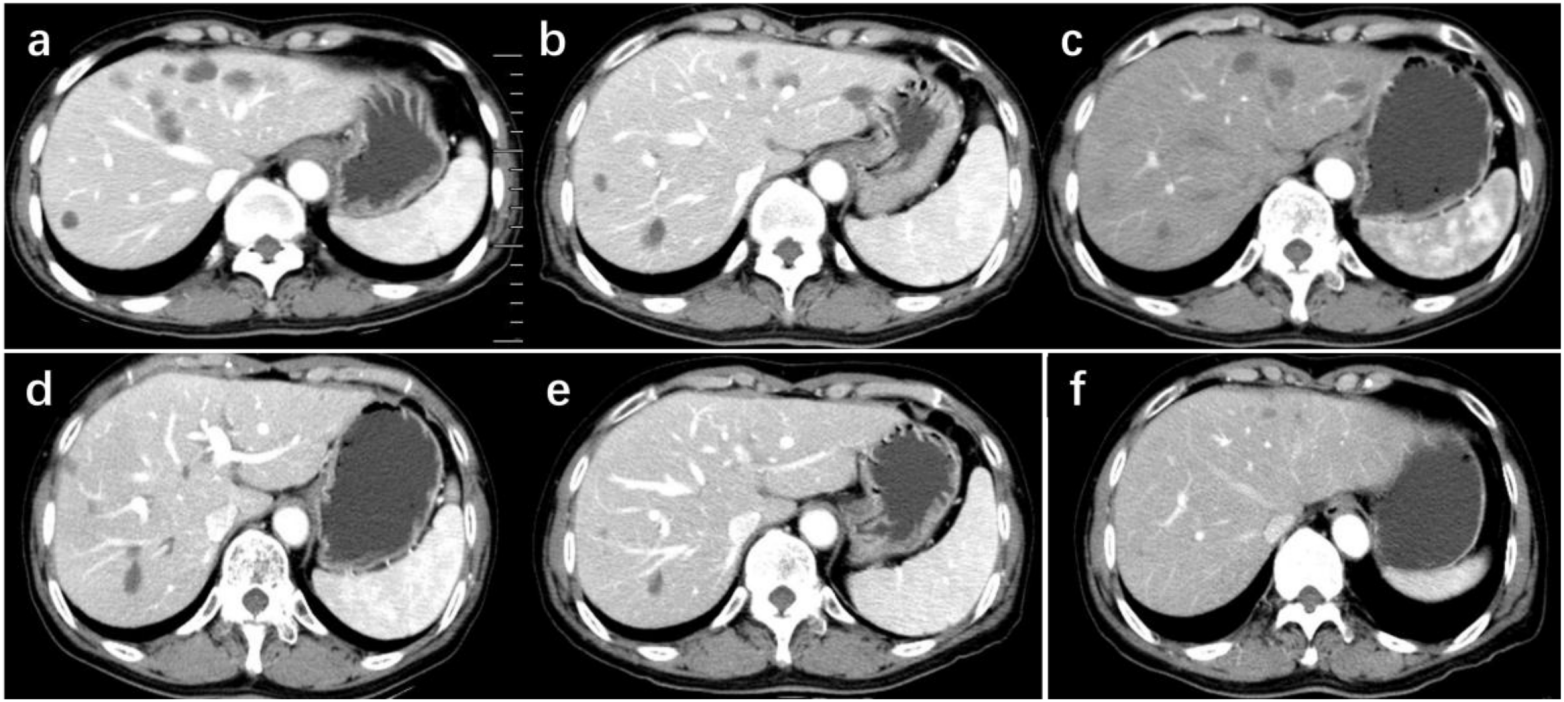
Serial contrast-enhanced CT scans demonstrated progressive reduction of hepatic metastatic lesions. The initial scans showed multiple billboard metastases on June 21, 2022 **(a)**, November 2, 2022 **(b)**, and January 5, 2023 **(c)**. Follow-up imaging on March 6, 2024 **(d)**, September 5, 2024 **(e)**, and April 9, 2025 **(f)** revealed marked regression with minimal residual abnormalities and near-complete radiologic remission.

The patient has maintained complete remission for more than 43 months with ECOG 0 performance status. Sequential CT evaluations demonstrated consistent shrinkage and long-term stabilization of hepatic metastases, with no new lesions detected throughout follow-up (**Figure 2**).

## Discussion

Gastric cancer often portends a very dismal prognosis, particularly in the immune-cold type of tumor, including HER2 negativity, PD-L1 CPS <1, microsatellite stability, and low tumor mutational burden (6, 8). Such biological characteristics are also linked to little immunosecheckpoint inhibitor monotherapy responsiveness and therefore novel treatment modalities are necessitated.

Early termination of FOLFOX in this case by intense toxicity necessitated the therapeutic priorities to be reviewed. It is now indicated by the work of CheckMate-649 and ORIENT-16 that a reduction in tumor burden following chemotherapy can sensitize later responsiveness to checkpoint blockade, even in PD-L1-negative illness (9, 10). Another theory that stimulates the initiation of immunotherapy under the low-disease-burden state is that of immunogenic cell death in facilitating antigen exposures and T-cell priming (11).

Envafolimab and S-1 were selected as they were selected because it was more tolerable and synergistic. Envafolimab is associated with a lower risk of infusion and a sustained chronologic relationship of subcutaneous PD-L1 inhibition and the development of S-1 shows 5-FU that is better tolerated with cytostatic control (12, 13). Also, it is hinted that fluoropyridines can help to enhance antitumor immunity, which supports that such a combination is biologically plausible (14, 15).

The remarkable long-term complete remission in this immune cold tumor means that special consideration should be given when formulating treatment. In patients selected who have succeeded in controlling disease by initial chemotherapy, maintenance immunotherapy administered with oral fluoropyrimidine can give long-term advantage without loss of quality of life.

Nevertheless, the lack of post-treatment biopsy and absence of Claudin18.2 testing limit deeper biological interpretation (16). Prospective studies are needed to determine which subgroups of immune-cold gastric cancer may derive the greatest benefit from maintenance strategies.

## Conclusion

This case demonstrates that individualized maintenance therapy combining envafolimab and S-1 can produce durable remission and long-term survival in metastatic gastric cancer with immune-cold characteristics. This strategy may benefit patients intolerant to intensive chemotherapy, though larger studies are needed to refine patient selection.

## Data Availability

The raw data supporting the conclusions of this article will be made available by the authors, without undue reservation.
