# Correction: Long-term survival of an advanced gastric cancer patient with multiple liver metastases following maintenance therapy with envafolimab and oral chemotherapy: a case report

**DOI:** 10.3389/fonc.2026.1798299

**Published:** 2026-02-10

**Authors:** Frontiers Production Office

**Affiliations:** Frontiers Media SA, Lausanne, Switzerland

**Keywords:** advanced gastric cancer, liver metastases, immunotherapy, envafolimab, raltitrexed, maintenance therapy, long-term survival

Affiliation “Zist Balin Co. Iran”, was erroneously given as “Sodour Ahrar Shargh Co., Iran”.

The original version of this article has been updated.

